# Pathogenic Characteristics of an Infection with Canine Influenza Virus and *Streptococcus equi* subsp. *zooepidemicus* Alone or in Combination in Mice

**DOI:** 10.1155/2024/2237621

**Published:** 2024-01-17

**Authors:** Dan Zhao, Yaru Sun, Jingjing Guo, Yuping Tang, Zhibo Wang, Xia Wen, Yuhao Dong, Yongjie Liu

**Affiliations:** Joint International Research Laboratory of Animal Health and Food Safety, College of Veterinary Medicine, Nanjing Agricultural University, Nanjing, Jiangsu, China

## Abstract

Both *Streptococcus equi* subsp. *zooepidemicus* (SEZ) and canine influenza virus (CIV) are two important pathogens causing infectious respiratory disease in dogs and are frequently codetected in respiratory secretions. However, the clinical significance of viral/bacterial coinfection remains unknown. This study investigated the pathogenic characteristics of infection with CIV and SEZ alone or in combination in mice. Our data indicated that the severity of the disease is related to the challenge order of CIV and SEZ. Coinfection of CIV and SEZ induced higher weight loss in mice than single infection, except for the VB group (viral followed by secondary bacterial infection). Compared with the concurrent or sequential infection groups of CIV and SEZ, mice in the CIV–SEZ preincubation group exhibited more obvious weight loss, higher mortality, and significantly enhanced burden of SEZ and CIV in tissues. Interestingly, viral and bacterial preincubation before coinfection caused typical pulmonary fibrosis in mice. Correspondingly, transforming growth factor (TGF)-*β* was upregulated, and its canonical small mother against decapentaplegic (Smad) 2/3 signaling was noticeably induced. Further investigation indicated that the activity of the viral neuraminidase (NA) enzyme upon sialic acid was considerably increased due to the direct interaction of CIV with SEZ, which may be related to the activation of the TGF-*β* signaling pathway. These findings implicate an unexpected contribution of the direct interaction between CIV and SEZ to synergistic pathogenicity.

## 1. Introduction

Canine infectious respiratory disease (CIRD) is an endemic disease caused by a variety of viral and bacterial pathogens. Common clinical symptoms include runny nose, cough, respiratory distress, fever, lethargy, infection of the lower respiratory tract, and even death [1]. The disease is highly contagious and often causes a collective infection where dogs are housed in groups or exposed to transient populations of other dogs, such as in kennels, dog shelters, or training bases. *Streptococcus equi* subsp. *zooepidemicus* (SEZ) is a zoonotic bacterium that is symbiotic in horses but highly pathogenic and lethal in other animals [2]. SEZ has been identified as the classic pathogenic agent of CIRD, and most infected dogs are classified as severe fibrin-suppurative, necrotizing, and hemorrhagic [3, 4]. This bacterium has caused several outbreaks of hemorrhagic pneumonia in dogs in recent years [5–7]. Influenza A virus (IAV), a segmented, negative single-stranded RNA virus of *Orthomyxoviridae*, is an important respiratory pathogen in dogs. Equine-origin H3N8 and avian-origin H3N2 canine influenza virus (CIV) may pose a public health threat [8, 9]. Weakened immune systems due to influenza are highly prone to secondary infections with other pathogens, leading to disease progression and even death [10, 11]. CIV and SEZ have been isolated from cases of canine respiratory disease, particularly in kennels and shelter dogs [12]. The severity of disease caused by CIV increased in the presence of SEZ [13], but the clinical significance of coinfection with the two pathogens remains unclear.

Previous studies with IAV have shown that its infection predisposes the host to secondary bacterial infection in a variety of ways, including destruction of the mucosal barrier or disorder of the immune responses [14–16]. In addition to the widely described immune modulation and tissue-remodeling mechanisms of bacterial–viral synergy, direct bacteria–virus interactions are also functional. Rowe et al. [17] found that the direct interaction between IAV and *Streptococcus pneumoniae* promoted bacterial adherence. Further, David et al. [18] reported that this direct interaction altered the host response to either individual pathogen, as when IAV-pneumococcus complexes were utilized as vaccine antigens, the efficacy was greater than that seen with either vaccine alone. Inspired by these studies, in this work, we test whether a direct partner relationship between CIV and SEZ exists. Our results demonstrated that the direct incubation of CIV with SEZ increased the colonization ability of either individual pathogen in murine tissues and caused aggravated pulmonary fibrosis in mice. We also found that the severity of the disease is related to the challenge order of CIV and SEZ. This work provides key results on the pathogenesis of CIRD caused by multipathogen infection.

## 2. Materials and Methods

### 2.1. Viral and Bacterial Strains

H3N2 influenza virus of A/Canine/Jiangsu/06/2010 (JS/10), GenBank number JN247616-JN247623, was isolated from nasal swabs of dogs with respiratory symptoms, and a mouse-adapted strain was prepared [19]. The titer of CIV was determined by plaque-forming unit (PFU) with Madin–Darby canine kidney cells [20]. Viruses were dispersed in phosphate-buffered saline (PBS) kept at −80°C.

SEZ strain BJ-1 was isolated from the lung of a dog that died of hemorrhagic pneumonia (GenBank number: PRJNA991107). The bacterial strain was grown in Todd–Hewitt broth supplemented with 1.5% (w/v) yeast extract at 37°C. Bacteria in the logarithmic phase were collected and washed three times with PBS and adjusted to the required concentration for the experiment. The colony forming units (CFU) of BJ-1 were determined by plating on a solid medium.

### 2.2. Animal Grouping

Mice are widely believed to be an ideal model for CIV [21–23] and SEZ [24–26]. One hundred forty mice (BALB/c, 5 weeks old, female) were randomly divided into seven groups, with 20 mice in each group: single bacterial infection (Group B), single viral infection (Group V), virus–bacteria sequential infection (Group VB), bacteria–virus sequential infection (Group BV), virus–bacteria simultaneous infection (Group CO), virus–bacteria preincubation before simultaneous infection (Group PRE) and PBS control (Group PBS). According to the protocol presented in Table 1, the mice under anesthesia with 4% isoflurane were infected with SEZ or CIV for the indicated time points by intranasal inoculation. Each challenge inoculum was 5 *×* 10^5^ CFU of bacteria and/or 5 *×* 10^5^ PFU of virus in 50 *μ*L of PBS and the amount indicated in CFU/mL or PFU/mL. This concentration of SEZ and CIV was selected on the basis of a preliminary sighting study, in which the starting concentration was screened from the levels of 1 × 10^5^, 5 × 10^5^, 1 × 10^6^, 5 × 10^6^, and 1 × 10^7^ CFU or PFU as a concentration expected to show obvious clinical symptoms but with a mortality rate below 20% after single or combined infection of the two pathogens. In the CO group, SEZ and CIV were mixed in 50 *μ*L of PBS and immediately used to infect mice. In the PRE group, the two pathogens were coincubated in PBS for 12 hr at 25°C before the challenge. To ensure comparability, pathogens in all groups were placed at *25*°C for 12 hr. Before infection, the viability of SEZ and CIV was performed by quantification with CFU or PFU and the amount expressed in CFU/mL or PFU/mL [20].

### 2.3. Clinical Monitoring and Sample Collections

Mice were weighed daily, and the body weight loss was calculated as the percentage with the starting weight. Four mice from each group were randomly selected and euthanized at 2, 4, 6, 10, and 14 days postinfection (dpi). Corresponding samples were collected according to the test requirements.

### 2.4. Measurement of Bacterial Load

Blood was collected retroorbitally and transferred to a blood collection tube containing EDTA to prevent clotting. Lung and brain tissues were obtained aseptically from euthanized mice. Four fecal particles were collected from the rectum at the front of the anus. All samples were weighed, homogenized, and then diluted multiple times before being spread onto THY culture plates. The bacterial count was subsequently normalized based on the weight of the sample and recorded as log10 CFU/g.

### 2.5. Measurement of Viral Load

Samples of lung, brain, blood, and feces were tested to determine the viral load. Viral RNA was extracted from samples using a viral nucleic acid extraction kit (T800, Tianlong Technology, Xi'an, China). According to the directions provided by the kit, viral nucleic acids can be extracted from various sample types, including tissues, whole blood, and feces. cDNA was synthesized using the primer Uni12 [27] (5′AGCAAAAGCAGG3′) with HiScript II Q Select RT SuperMix for qPCR (+gDNA wiper) (Vazyme, Nanjing, China). Absolute RT-qPCR was performed on a 7300 Real-Time PCR System (Applied Biosystems, Foster City, California, USA). The detection primers were designed for matrix (M) protein (06MF/R: 5′TCTATCGTCCCATCAGGC3′/5′GGTCTTGTCTTTAGCCATTC3′). The standard was a recombinant plasmid constructed by inserting the virus M gene into the multiple cloning site of the pMD19-T plasmid (TaKaRa, Dalian, China), and the initial concentration of the standard was set to 100 ng/*μ*L and diluted with deionized water. The dilution from 10^−2^ to 10^−9^ of the standard was tested with samples for quantification. The standard curve was drawn according to the corresponding relationship between the copy number and cycle threshold. The copy number per gram was calculated and recorded as log10 copies/g. The conversion between copy number and DNA concentration was given by the formula copies/mL = (6.02 × 10^23^ × (concentration ng/*μ*L × 10^−9^))/(DNA full length × 660) [28].

### 2.6. Hematoxylin and Eosin (H&E) Staining and Masson Staining

The lung and brain tissues at 6 dpi were removed intact, and any blood on the tissue surface was washed off using PBS. The tissues were then fixed in 4% paraformaldehyde. Histopathology was performed using H&E staining. The tissue sections were blindly examined and graded based on the pathological features observed in the lesions [29]. A score <1 indicates mild pathological damage, 1–3, not including 3, indicates moderate damage and 3 or higher indicates severe damage. To analyze the degree of fibrosis, Masson staining was performed on lung tissue sections. Collagen deposition was demonstrated by blue staining, and muscle fibers were stained red. Ashcroft scores were used to evaluate pathological changes and the degree of pulmonary fibrosis [30]. A score <1 indicated no or mild fibrosis, 1–3, not including 3, indicated moderate fibrosis, and 3 or higher was considered typical fibrosis.

### 2.7. Measurement of Cytokine mRNA Expression Levels

Total RNA from the lung and brain was extracted using a total RNA extraction kit (Omega Bio-Tek, GA, USA). Total RNA was reverse transcribed into cDNA using HiScript II Q RT SuperMix for qPCR (+gDNA wiper) (Vazyme, Nanjing, China). The relative transcription levels of interleukin-6 (IL-6) (IL-6F/R: 5′CCACTTCACAAGTCGGAGGCTTA3′/5′GCAAGTGCATCATCGTTGTTCATAC3′), tumor necrosis factor-*α* (TNF-*α*) (TNF-*α*F/R: 5′AAGCCTGTAGCCCACGTCGTA3′/5′GGCACCACTAGTTGGTTGTCTTTG3′), interferon-*β* (IFN-*β*) (IFN-*β*F/R: (5′ATGACCAACAAGTGTCTCCTCC3′/5′GCTCATGGAAAGAGCTGTAGTG3′) and transforming growth factor-*β* (TGF-*β*) (TGF-*β*F/R: 5′AATGGTGGACCGCAACAAC3′/5′GCACTGCTTCCCGAATGTC3′) were measured. The reference gene was glyceraldehyde-3-phosphate dehydrogenase (GAPDH) (GAPDH F/R: 5′CATCTTCCAGGAGCGAGACCC3′/5′TTTCTCGTGGTTCACACCCAT3′). Relative transcription levels of cytokines were calculated using the 2^−*ΔΔ*CT^ method [31].

### 2.8. Immunofluorescence Staining

Lung tissue sections were utilized for conducting indirect immunofluorescence assays, and immunofluorescence staining of the sections was performed as described previously [32]. Sections were placed in a boiling antigen repair solution (1 mmol Tris-EDTA, pH = 9.0) and boiled for 15 min, kept warm for 15 min for antigen repair, and then blocked with 5% bovine serum albumin (BSA) at 37°C for 1 hr. Rabbit antiphosphor-small mother against decapentaplegic (Smad) 2/3 (Thr8) antibody (1 : 2,000, Bioss, Beijing, China) were added after drying the blocking solution and incubated for 1 hr at 37°C. The sections were washed three times with PBS for 3 min each time. Subsequently, fluorescently labeled goat antirabbit IgG (1 : 5,000, Abcam, Cambridge, Britain) was added and incubated at 37°C for 1 hr. Tissues were washed three times with PBS, and special attention was given to avoid light exposure throughout the incubation to prevent fluorescence quenching. One hundred microliters of an antifluorescence quenching tablet (Biosharp, Guangzhou, China) were added to each slice. Finally, the stained sections were observed and photographed for analysis using a fluorescence microscope.

### 2.9. SDS‒PAGE and Western Blot

To measure protein expression levels in lung tissues, the tissues homogenated in RIPA lysate (containing protease and phosphatase inhibitors) (Sangon Biotech, Shanghai, China) were centrifuged at 12,000 rpm for 3 min and the supernatant was collected. The protein concentration in the supernatant was determined using a BCA protein assay kit (Thermo Fisher, Waltham, MA, USA). Equal amounts of protein with SDS-loading buffer were loaded onto SDS‒PAGE gels for separation. The protein from the gel was transferred onto a PVDF membrane (Millipore, Boston, USA). To prevent nonspecific binding, the membrane was blocked using 5% BSA. The membrane was then incubated with the primary antibody overnight at 4°C. The primary antibody used here was a rabbit anti-phospho-Smad 2/3 (Thr8) (Bioss) diluted with PBS at 1 : 2,000. The membrane was washed with PBST, and goat antirabbit IgG H&L (HRP) secondary antibody (Biosharp) diluted with PBS at 1 : 5,000 was added and incubated at room temperature for 1 hr. The unbound antibody was washed off with PBST, and ECL femto light chemiluminescence solution (Epizyme, Shanghai, China) was added for exposure (Bio-Rad, California, USA). Protein bands were analyzed with ImageJ for quantitation, and GAPDH was used as the control.

### 2.10. Statistical Analyses

GraphPad Prism 8 software (GraphPad Software Inc., California, USA) was used for data analysis. Data were shown as the mean with SEM, max, and min (bacterial load), and analysis of variance was performed with ANOVA, Kaplan–Meier was used to analyze the survival curve. *P* < 0.05 indicated a significant difference.

## 3. Results

### 3.1. Coinfection with SEZ and CIV Causes Severe Symptoms in Mice, but Sequential CIV/SEZ Infection Does Not

**Figure 1:**
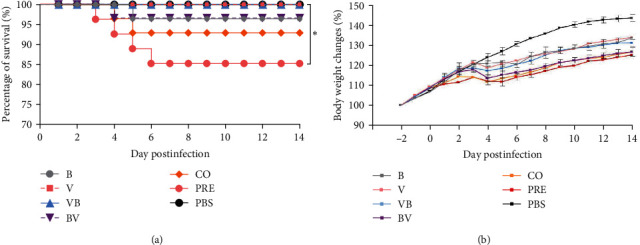
Survival curves (a) and body weight changes (b) in mice. (a) Survival curves of mice.  ^*∗*^*P*  < 0.05 indicates that the PRE group is significantly different from the V, VB, and PBS groups. (b) Body weight changes. Mice were monitored throughout the observation period for 14 days, and the curve was portrayed as the percentage of the starting weight.

As depicted in Figure 1(a), no deaths of mice were observed in the V, VB, and PBS groups. In the other groups, one to four mice succumbed to the infection. Importantly, the mortality rate of mice in the PRE group exhibited statistically significant differences compared to the V, VB, and PBS groups (*P* < 0.05).

Body weight changes in mice are shown in Figure 1(b), and the significance analysis is shown in Table 2. Mice in each infection group showed a certain degree of weight gain deceleration or weight loss from 2 to 6 dpi. In general, mice infected with SEZ alone (Group B) exhibited the largest weight loss at 6 dpi, and mice infected with CIV alone (Group V) exhibited the largest weight loss at 4 dpi. Mice infected with SEZ followed by secondary CIV infection (Group BV) showed more significant weight loss than those in the B, V, and VB groups, but compared with the CO and PRE groups, no significant difference was observed. These data suggest that coinfection of SEZ and CIV causes severe symptoms in mice, especially in mice infected with the preincubation pathogens, which exhibited the highest mortality. Furthermore, the data also suggest that CIV/SEZ sequential infection does not cause severe symptoms in mice.

### 3.2. Coinfection with CIV Significantly Increases the Bacterial Load in Mouse Tissues and Fecal Shedding, but CIV/SEZ Sequential Infection Does Not

To determine whether there were differences in bacterial load and shedding between groups, the counts of SEZ in lung, brain, blood, and feces were determined.

**Figure 2:**
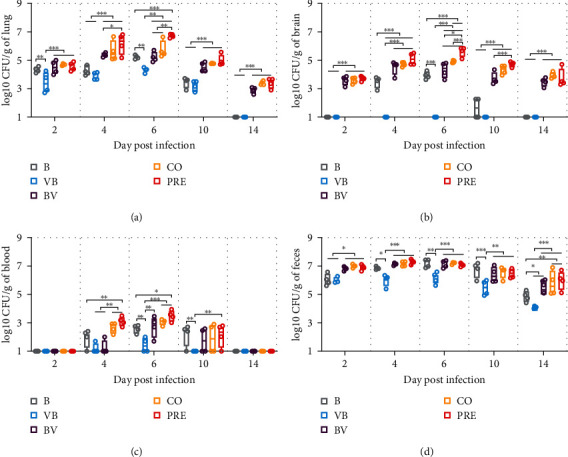
Bacterial load in mice. (a–d) Bacterial load of lung, brain, blood, and feces samples. Data are expressed as Max and Min.  ^*∗*^*P*  < 0.05,  ^*∗∗*^*P*  < 0.01,  ^*∗∗∗*^*P*  < 0.001. Samples with homogenized and gradient dilution were coated on THY plates. After overnight culture, single colonies were counted, and bacterial loads were normalized as log10 CFU/g.

The SEZ burden of lung tissues (Figure 2(a)) in each group reached the highest level at 6 dpi, with a load between 4.5 log10 CFU/g and 6.5 log10 CFU/g. Of all groups, the load in the VB group was consistently lower than that in the other groups and was significantly lower than that in Group B at 2 and 6 dpi (*P* < 0.01). The loads in the BV, CO, and PRE groups were higher at each time point, cleared more slowly than those in the B and VB groups, and were significantly different from those in the B and VB groups from 4 to 10 dpi (*P* < 0.01 or *P* < 0.001). The load in the PRE group was significantly higher than that in the CO group at 6 dpi (*P* < 0.01).

The results of bacterial load in brain tissue (Figure 2(b)) showed that no bacteria were detected in the brains of mice in the VB group, but the bacterial load in the brains of mice in the BV group was significantly higher than single B group (*P* < 0.001), indicating that mice preexposed with CIV did not promote SEZ colonization in brain tissues, but secondary infection with CIV did. The bacterial load in CO and PRE groups was always higher than single and sequential infection groups, and there was a significant difference on 6 dpi, and on that day, SEZ load in PRE was significantly higher than CO (*P* < 0.001). These results suggest that post-infection or coinfection with CIV can promote the colonization of SEZ in brain tissues.

SEZ infection alone and sequential or simultaneous infection with CIV caused bacteremia in mice (Figure 2(c)), but bacteremia was discovered from 4 to 10 dpi. Bacterial loads were all within 4 log10 CFU/g, and bacteria could not be detected in the blood of all mice. The bacterial load of blood in the PRE group was significantly higher than that in the B group from 4 to 6 dpi (*P* < 0.05 or *P* < 0.01), but there was no significant difference between the B group and the other groups. However, a shorter bacterial detection time and a significantly lower load in the VB group were found, and the SEZ load of VB was significantly lower than that of all groups, including the B group, from 6 to 10 dpi (*P* < 0.01 or *P* < 0.001). These data show that mice infected with CIV prior to SEZ were less likely to develop bacteremia.

The fecal bacterial load (Figure 2(d)) showed that the BV, CO, and PRE groups did not differ in fecal bacterial shedding, which was higher than that in the B and VB groups. In terms of fecal output, the VB group was always the least and significantly lower than all groups, including B, from 4 to 14 dpi (*P* < 0.05).

All results of bacterial load and shedding indicate that preincubation with CIV improves the colonization of SEZ in lung and brain tissues, but mice preposed to CIV do not promote but rather prevent the colonization of SEZ.

### 3.3. Coinfection with SEZ Has Little Effect on the Viral Load in Mouse Tissues, but Infection with CIV–SEZ Preincubation Is an Exception

To better understand viral load and fecal shedding in mice, viral RNA was quantified by absolute RT-qPCR in lung, brain, blood, and feces from mice euthanized at 2, 4, 6, 10, and 14 dpi.

**Figure 3:**
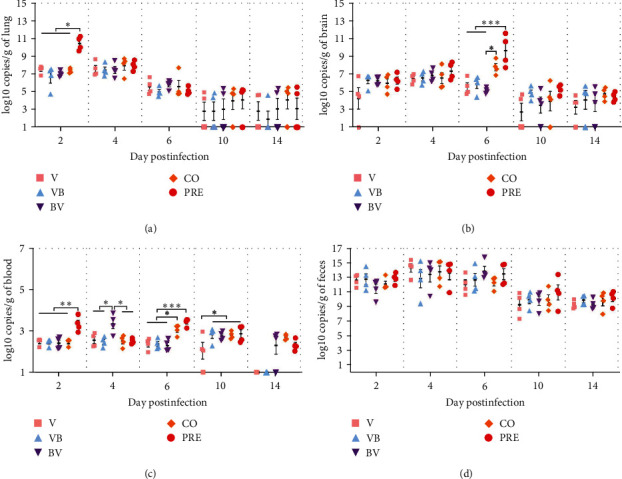
Viral load in mice. (a–d) Viral load of lung, brain, blood, and feces samples. Data are expressed as the means ± SEMs,  ^*∗*^*P*  < 0.05,  ^*∗∗*^*P*  < 0.01,  ^*∗∗∗*^*P*  < 0.001. The viral RNA in homogenized samples was extracted, and the amount of the M gene was measured by RT-qPCR with absolute quantification. The viral load was calculated by referring to the standard curve and expressed as log10 copies/g.

The lung viral loads (Figure 3(a)) showed that the load in the lungs of each group was high, from 2 to 4 dpi, and then gradually decreased over time. At 2 dpi, the maximum viral load of the lung in the PRE group reached 11 log10 copies/g, which was significantly higher than that in the V, VB, BV, and CO groups (*P* < 0.05).

In brain tissues (Figure 3(b)), it was shown that the viral load of mice in the PRE group was significantly higher than that in all groups at 6 dpi (*P* < 0.001), except for CO, and there was a significant difference between the CO and BV groups (*P* < 0.05). In addition, at other dpi, there was no significant difference in viral load among each group.

The viral load in blood (Figure 3(c)) showed that the viral content in blood was approximately 2–4 log10 copies/g. Because the low load was close to the detection line, we think the potential significance of one star is not significant. At 2 and 6 dpi, the load of the PRE group was significantly higher than that of the single and secondary infection groups (*P* < 0.01 or *P* < 0.001). These results indicate that the load of CIV increases under preincubation with SEZ *in vitro*.

The fecal viral load results (Figure 3(d)) showed that all infected mice excreted a large number of viruses through feces, and there was no difference in viral excretion between all groups.

### 3.4. Coinfection with SEZ and CIV Aggravates Lung Lesions, with Evident Pulmonary Fibrosis upon Simultaneous Infection

Considering that the symptoms of affected mice were most obvious at 6 dpi, the gross lung lesions, and tissue pathological changes on that day were observed to analyze the degree, type, and score of lesions of the different infection groups.

**Figure 4:**
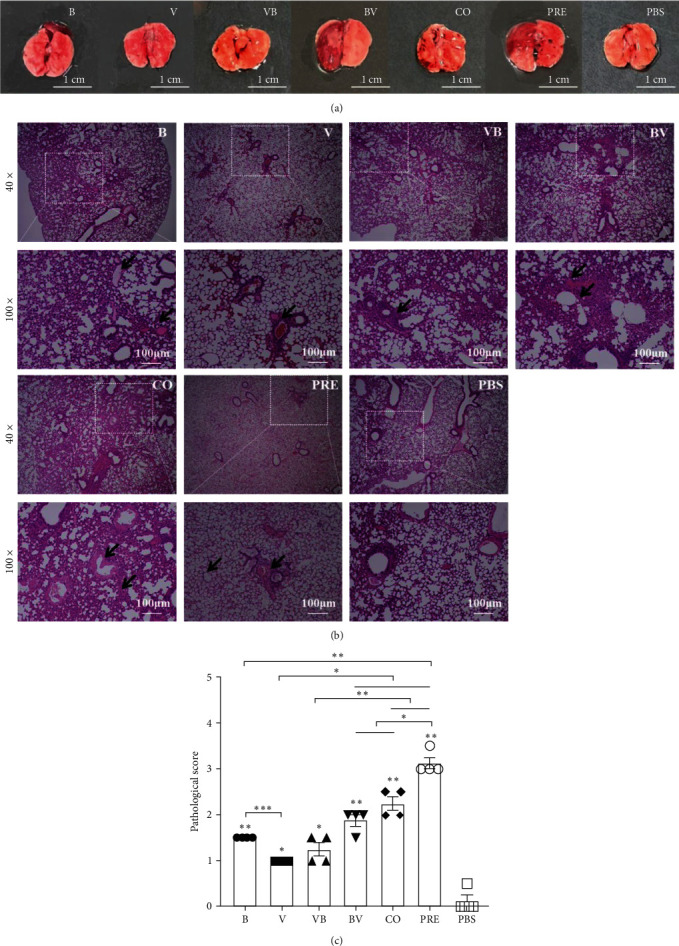
Gross pathology and histopathology of the lung tissues in mice. (a) Gross pathology of lung tissues. The scale bar is 1 cm. (b) Histopathological lesions of the lung. The scale bar is 100 *μ*m. Arrows indicate lesion areas. (c) Pathological scores of lung tissues. Five random areas in each group of lung tissue sections were blindly examined for the extent and grade of lesions. A score <1 indicates mild pathological damage, 1–3, not including 3, indicates moderate damage, and 3 or higher indicates severe damage.  ^*∗*^*P*  < 0.05,  ^*∗∗*^*P*  < 0.01, and  ^*∗∗∗*^*P*  < 0.001.

The results of gross lung lesions (Figure 4(a)) showed that all the infected mice exhibited hemorrhage and necrosis, although to varying degrees. The lungs in Groups B, BV, and PRE also experienced more obvious swelling. Compared with Group B, the areas of hemorrhage and necrosis in Groups BV, CO, and PRE were larger.

Pathological observation showed different levels of pneumonia in infected mice (Figure 4(b)). In the mice from Group B, most of the alveolar walls were significantly thickened, and the alveolar cavity was filled with a large amount of pale pink serous fluid, accompanied by a small amount of cellulose exudation. Mice in Group V showed a few alveolar walls thickened with light pink serous fluids exuded from the alveolar cavity, accompanied by a small amount of cellulose exuded, and part of the bronchi and bronchioles filled with erythrocytes and slight neutrophils. In the VB group, red blood cells and serous fluid infiltrated some pulmonary veins and pulmonary arteries, and a small amount of light pink serous fluid and cellulose exuded from the alveolar cavity. In the BV group, the blood vessels in alveolar walls were obviously dilated and engorged, part of the alveolar walls were damaged, and a large number of exudates, mainly composed of neutrophils and erythrocytes, filled the alveolar spaces. Lungs in the CO group showed significant hyperplasia in alveolar walls, the alveolar basement membrane and bronchiolar walls were covered with homogeneous, amorphous eosinophilic transparent membranes, and a large number of erythrocytes and a small amount of inflammatory cells infiltrated the bronchi and bronchioles. The main feature of the lungs in the PRE group was that the alveolar cavity was filled with fibrin exudates, accompanied by a large number of red blood cells and inflammatory cells.

Five random areas in each group of lung tissue sections were blindly examined (Figure 4(c)). The lesion areas and scores of Groups V and VB were the lightest, while the lesions of Groups B, BV, CO, and PRE successively increased, and the extent and area of group PRE were the most of all groups (*P* < 0.05 or *P* < 0.01).

**Figure 5:**
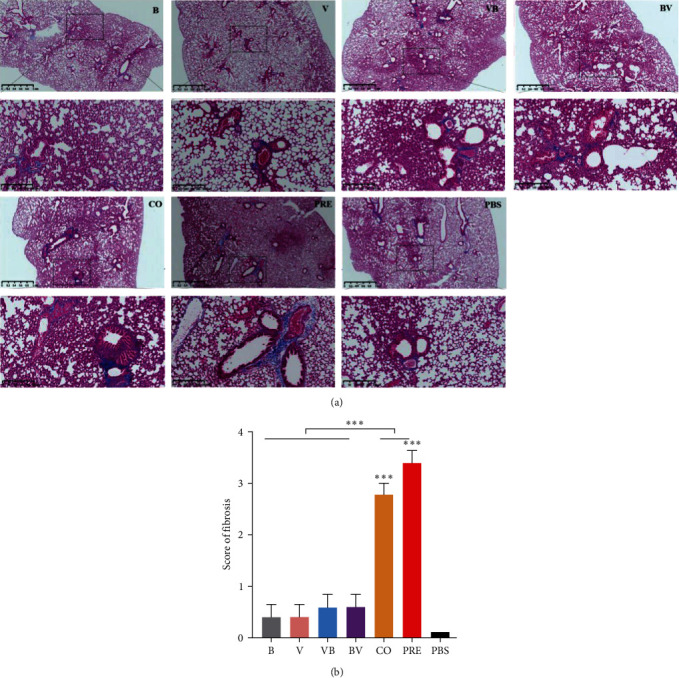
Histological analysis of the severity of lung fibrosis in mice. (a) Representative images of Masson staining. (b) Quantitative mean score of the severity of fibrosis. The Ashcroft score of fibrosis was determined by a trained pathologist blinded to the experimental groups.

To further determine the degree of fibrosis, Masson staining was performed on the lung sections (Figure 5(a)); concurrent infection with SEZ and CIV, regardless of whether the two pathogens had preincubated before infection, induced more fibrosis than the other treatments at 6 dpi. This result was further confirmed in subsequent scoring (Figure 5(b)), which indicated a higher fibrosis level (*P* < 0.001) in the CO and PRE groups. There was no statistical difference between the CO and PRE groups.

### 3.5. Simultaneous Infection with SEZ and CIV Causes Obvious Meningitis in Mice

**Figure 6:**
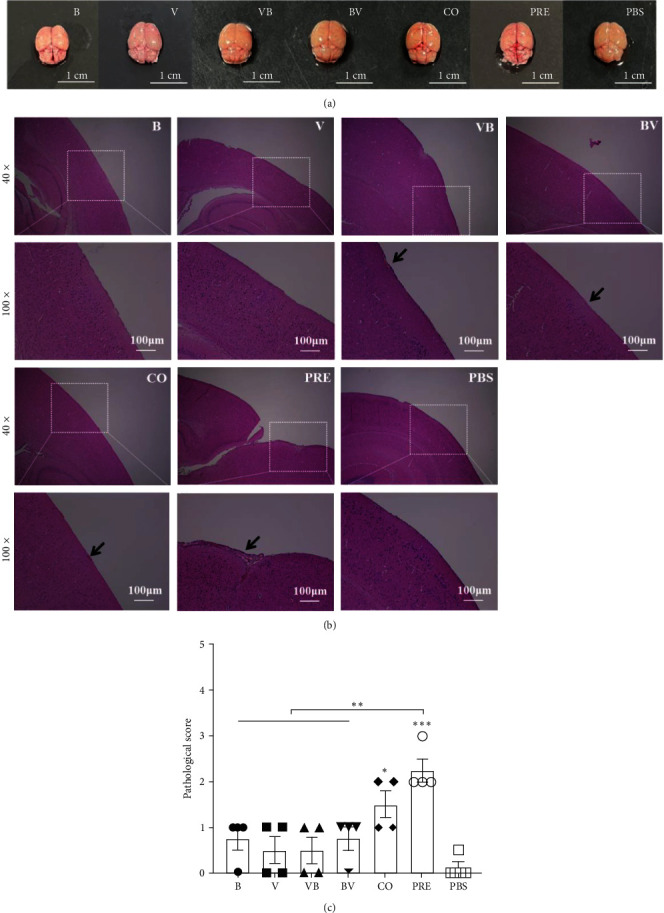
Gross pathology and histopathology of the brain tissues in mice. (a) Gross pathology of brain tissues. The scale bar is 1 cm. (b) Histopathological lesions of the brain. The scale bar is 100 *μ*m. Arrows indicate lesion areas. (c) Histopathological scores of the brain. A score <1 indicates mild pathological damage, 1–3, not including 3, indicates moderate damage, and 3 or higher indicates severe damage.  ^*∗*^*P*  < 0.05,  ^*∗∗*^*P*  < 0.01, and  ^*∗∗∗*^*P*  < 0.001.

The results of brain anatomical changes (Figure 6(a)) showed that the brain surface of mice in the PRE group showed relatively obvious vascular congestion, and other groups did not differ from PBS at the naked eye level. A few spotty hemorrhages on the meninges were observed in the lungs of the VB, BV, and CO groups, and leptomeningitis was the cardinal feature in mice of the PRE group in pathological sections (Figure 6(b)). Therefore, only mice in the CO and PRE groups had moderate brain damage (Figure 6(c)). These results confirm that simultaneous infection with SEZ and CIV aggravates histopathology in mice.

### 3.6. Simultaneous Infection with SEZ and CIV Induces Significantly Higher Expression Levels of TNF-*α*, IL-6, IFN-*β*, and TGF-*β* in the Lung and Brain

To explore the difference in cytokine levels induced by viruses and bacteria alone or in combination, we measured the transcription levels of TNF-*α*, IL-6, IFN-*β*, and TGF-*β* in the lung and brain.

**Figure 7:**
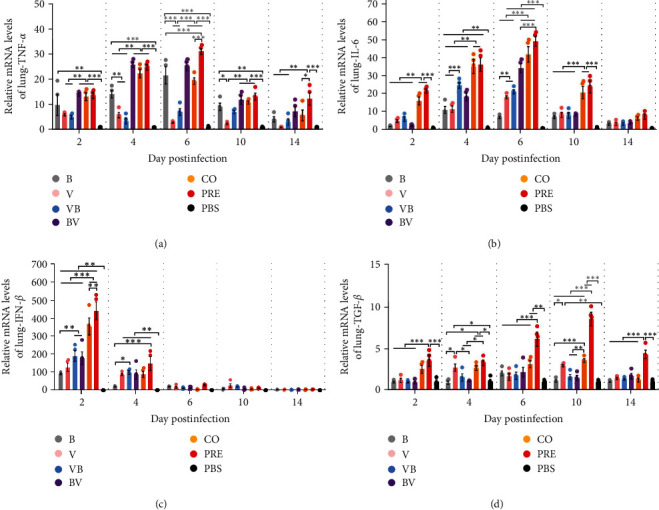
Transcription levels of cytokines in lung tissues. (a–d) Transcription levels of TNF-*α*, IL-6, IFN-*β*, and TGF-*β* in the lung. Total RNA was extracted from lung tissues, and relative RT-qPCR detection was performed for each cytokine gene. GAPDH was the housekeeping gene, and the results were expressed as 2^−*ΔΔ*CT^.  ^*∗*^*P*  < 0.05,  ^*∗∗*^*P*  < 0.01,  ^*∗∗∗*^*P*  < 0.001.

The mRNA transcription level of TNF-*α* in lung tissues (Figure 7(a)) showed that the TNF-*α* transcription level was significantly upregulated in Group B and was statistically significant compared with PBS from 2 to 10 dpi (*P* < 0.01 or *P* < 0.001). Although TNF-*α* in mice of Group V was upregulated 5–8 times at 2 and 4 dpi, it was not statistically significant compared with PBS. The situation in the VB group was similar to that in the V group. In the BV, CO, and PRE groups, relatively high TNF-*α* mRNA levels were measured, and there was no significant difference between the three groups at 2, 4, and 10 dpi. The trend of TNF-*α* was basically consistent with the change in bacterial load, which further indicated that the mRNA level of TNF-*α* was related to the load of SEZ.

The mRNA expression level of IL-6 in the lung (Figure 7(b)) showed that mice infected with SEZ alone (Group B) did not exhibit strong IL-6 expression and only upregulated significantly at 4 dpi, up to 11–20 times (*P* < 0.01) compared with PBS. The mRNA expression level of IL-6 in Group V increased by more than 15 times at 4 and 6 dpi, which differed from that in the PBS group (*P* < 0.01 or *P* < 0.001). In the secondary infection group, the mRNA expression level of IL-6 in VB was significantly higher than that in B and V at 4 dpi (*P* < 0.001), and that in BV was significantly higher than that in B and V at 6 dpi (*P* < 0.001). Compared with the B and V groups, the expression of IL-6 in the coinfection group (CO and PRE) from 2 to 10 dpi was significantly increased (*P* < 0.01), but there was no difference between the two. These results indicate that the expression of IL-6 is upregulated in the secondary and coinfection groups.

The mRNA expression level of IFN-*β* in the lungs (Figure 7(c)) indicated robust responses in each group, with a significant upregulation of 100- to 500-fold. However, the duration of these heightened responses was short-lived. Mice infected with a single pathogen (B or V) exhibited the lowest upregulation of IFN-*β*, ranging between 100 and 150-fold increase. Mice infected with two pathogens sequentially (VB and BV) demonstrated intermediate levels of IFN-*β* upregulation, and mice in the coinfection groups (CO and PRE) displayed the highest transcriptional upregulation of IFN-*β*.

The mRNA expression of TGF-*β* (Figure 7(d)) in the lungs was not significantly different in mice in the single or secondary infection groups compared with mice in the PBS group, except in Group V at 4 dpi (*P* < 0.05) and 10 dpi (*P* < 0.01). The transcription of TGF-*β* in mice in the CO and PRE was upregulated, and the mRNA expression level of TGF-*β* in mice in the PRE was much higher than that in mice in the CO from 6 to 10 dpi (*P* < 0.001).

**Figure 8:**
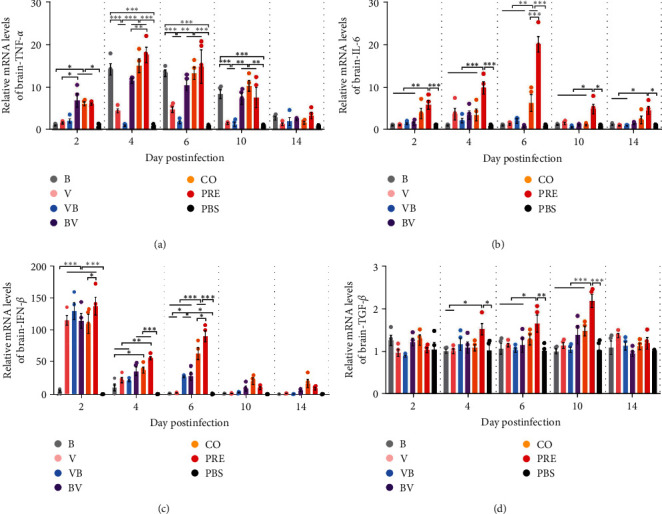
Transcription levels of cytokines in brain tissues. (a–d) Transcription levels of TNF-*α*, IL-6, IFN-*β*, and TGF-*β* in the brain. The results were expressed as 2^−*ΔΔ*CT^.  ^*∗*^*P*  < 0.05,  ^*∗∗*^*P*  < 0.01,  ^*∗∗∗*^*P*  < 0.001.

The trend of the mRNA expression level of TNF-*α* in the brain (Figure 8(a)) was similar to that in the lung, but the fold change was below 22, which was lower than the value in the lung.

The mRNA expression level of IL-6 in the brain (Figure 8(b)) showed that only IL-6 in the CO and PRE groups was significantly upregulated, and the upregulation in the PRE group was 2–3 times higher than that in the CO group at 6 dpi (*P* < 0.001). In addition to 6 dpi, the mRNA expression level of IL-6 was significantly different from PBS only in the PRE group within the time of infection.

The mRNA expression level of IFN-*β* in the brain (Figure 8(c)) showed that on 2 dpi, the IFN-*β* level in all groups except B had a significant difference with PBS, but it is notable that on that day, neither bacteria nor virus was detected in the brains of mice in Group B. The upregulation level in IFN-*β* of BV, CO, and PRE groups at 4 dpi was significantly higher than in PBS (*P* < 0.001). At 6 dpi, abundant IFN-*β* expression could be measured only in the CO and PRE groups, and the difference between the two groups was significant (*P* < 0.05).

The expression of TGF-*β* (Figure 8(d)) in the brain was different from that in the lung. From 4 to 10 dpi, only the increase in PRE was significantly different from that in PBS, but the upregulation was only approximately 2 times, forming a significant difference compared with the other groups (4 dpi, PRE vs. B or V; *P*  < 0.05; 6 dpi, PRE vs. B, V, VB or BV; *P* < 0.05; 10 dpi, PRE vs. B, V, VB, BV or CO; *P* < 0.001).

The above cytokine data of lung and brain tissues suggest that simultaneous infection with SEZ and CIV induces significantly higher expression levels of cytokines in the lung and brain.

### 3.7. Simultaneous Infection with SEZ and CIV Significantly Activates the TGF-*β*/Smad Pathway in the Lung

**Figure 9:**
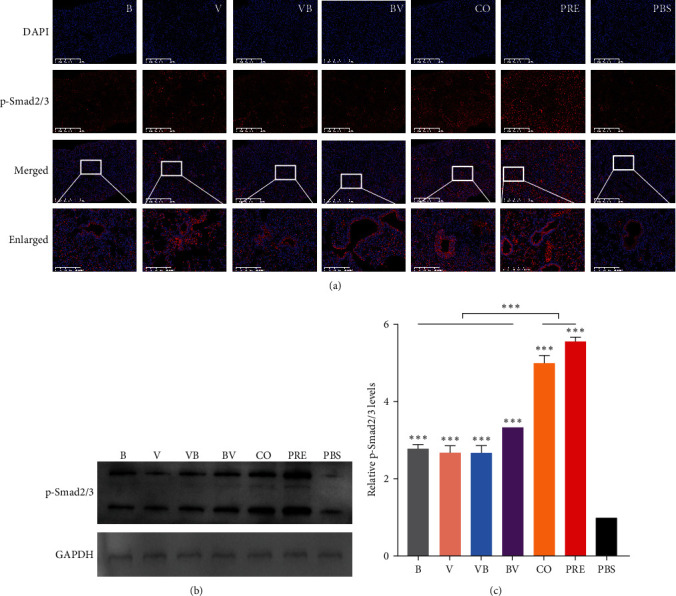
Downstream protein of TGF-*β* signal. (a) Indirect immunofluorescence assay of p-Smad2/3 proteins in lung tissues. DAPI staining of the nucleus is shown in blue, and p-Smad2/3 proteins are shown in red. The enlarged areas are shown as the representative sites of deposition with p-Smad2/3 proteins. (b) The expression of p-Smad2/3 proteins in lung tissue was detected by Western blot. (c) Band intensities relative to PBS were analyzed.  ^*∗*^*P*  < 0.05,  ^*∗∗*^*P*  < 0.01,  ^*∗∗∗*^*P*  < 0.001.

TGF-*β* exists in the cell matrix in a latent state and must be activated to exert biological functions. In particular, phosphorylated Smad2/Smad3 (p-Smad2/3) proteins are considered to be the key mediators of TGF-*β* signaling in tissue fibrosis and tumorigenesis [33]. Therefore, we detected p-Smad2/3 protein after activation of the TGF-*β* pathway. Immunofluorescence staining (Figure 9(a)) showed that p-Smad2/3 protein was mainly located in the bronchial bottom of mice in CO and alveolar cavities of mice in PRE. p-Smad2/3 quantitation in lung tissues (Figure 9(b)) and relative expression levels (Figure 9(c)) showed that compared with the PBS group, the expression level of p-Smad2/3 proteins in mice in each infection group increased to a certain extent, but only in the CO and PRE groups, and the expression level of p-Smad2/3 proteins in the lung was significantly higher than in the other groups, confirming the activation of the TGF-*β*/Smad pathway in mice with simultaneous SEZ and CIV.

## 4. Discussion

Coinfection of individual hosts by multiple pathogens is a pattern that is very commonly observed in natural populations. It is generally accepted that microbial coinfection increases the risk of disease severity in humans and animals. Traditionally, the reasons for coinfection to aggravate the disease include increased exposure to cell receptors [34], damage to the mucosal barrier [35, 36], and immune abnormalities during viral infection followed by secondary bacterial infection [37–39]. As common causative agents of CIRD, SEZ and CIV have many opportunities to coexist in the same infectious niche. They might be simultaneously or sequentially shed in the oral and nasal secretions or feces, with a high probability of direct contact with each other for a considerable period of time before spreading to the host. We hypothesize that the direct interaction between the two pathogens can influence pathogenic behavior such as virulence or confer a fitness advantage during their colonization or infection of the respiratory tract. Such behavioral influences have recently been demonstrated to occur between IAV and *S. pneumonia* [17]. The direct interaction enhances the initial colonization of pneumococcus and invasive disease in mice, while IAV directly benefits from this interaction by promoting stability and infectivity [40]. Consistent with these studies, our work found that compared with single or sequential infection of CIV and SEZ, CIV–SEZ concurrent infection caused more obvious weight loss, higher mortality, and significantly enhanced bacterial/viral burden and shedding in mice. It is interesting to note that the above changes were more pronounced in the CIV–SEZ preincubation group than in the mice coinfected with the two pathogens in the absence of preincubation. This finding indicates that the *in vitro* preincubation might alter the pathogenic characteristics of CIV or SEZ and thus exacerbate the coinfection. To exclude the possibility that the preincubation of SEZ with CIV affects the bacterial and viral infectious doses, we measured the content of CIV and SEZ after preincubation and demonstrated that preincubation did not exert any impact on the quantity of bacteria and viruses (*Supplementary 1*).

In this study, we found that CIV infection did not promote secondary bacterial infection; in contrast, it inhibited bacterial colonization in tissues, which seems to contradict earlier studies indicating that infection by a primary viral pathogen predisposed animals to secondary infection [41, 42]. We hypothesize that viral infection might improve the immunity of the host and strengthen a greater immune defense against bacterial invasion. During viral infection, type I interferon (IFN-I) is induced and involved in the innate antiviral response. Although less is known about the role of IFN-I in bacterial infections than in viral infections, IFN-I signaling has been reported to be involved in host defense by participating in the modulation of systemic inflammation against some bacteria, including *Streptococcus agalactiae, S. pneumonia*, and *Escherichia coli* [43]. Our data showed that the IFN-*β* mRNA level was significantly upregulated at the early stage of CIV infection. Therefore, it might be plausible that elevated IFN-*β* is a key player in the body's defense against SEZ infection. However, there is also a study indicating that influenza-induced IFN-I sensitizes hosts to secondary bacterial infections [44]. We speculate that the possible reasons for the differences are due to the level, timing, and ratio of IFN-I. Shepardson et al. [45] demonstrated that IFN-I expression can modulate susceptibility to methicillin-resistant *Staphylococcus aureus* (MRSA) infection, with IFN-*β* reducing host susceptibility to MRSA infection while IFN-*α* increases susceptibility.

Viruses may also contribute to elevated bacterial colonization. In this study, we found that subsequent CIV challenges after SEZ infection caused an increase in bacterial carriage. We speculate that viral presence might promote bacterial infection by facilitating bacterial attachment to host cells. Free CIV virions might bind directly to SEZ, thereby increasing bacterial proximity to the epithelial monolayer and augmenting attachment to host cell receptors. It has been shown that swine influenza virus (SIV) interacts directly with *Streptococcus suis* serotype 2, and the direct interaction is mediated by the hemagglutinin (HA) of SIV, which recognizes the *α*2,6-linked sialic acid present in the capsular polysaccharide of *S. suis* [46]. Additionally, we found that secondary viral infection caused a significant upregulation of TNF-*α*, IL-6, and IFN-*β* in lung and brain tissues compared to bacterial or viral infections alone. This result is logical, as the signaling pathways triggered by each pathogen can lead to synergistic stimulation of inflammation during coinfection. An overly robust inflammatory response could theoretically lead to lung injury without effectively clearing bacterial pathogens.

A previous study showed that bacterial lipopolysaccharide and peptidoglycan enhance the thermal stability of reovirus, leading to increased virus invasion [47]. Our study revealed that coinfection with SEZ increased the NA activity of CIV (*Supplementary 2*). NA activity is required for influenza viruses to release newly synthesized viruses by cleaving sialic acid both from host cell glycoconjugates and from oligosaccharides of viral HA and NA [48]. This action of NA also promotes adherence and invasion of *S. pneumoniae*, since the cleavage of sialic acid from the cell surface by NA exposes cryptic receptors for this bacterium [49]. Therefore, we speculate that enhanced NA activity induced by coinfection with SEZ may play an important role in viral or bacterial colonization. However, this requires further investigation.

One of the most interesting and striking patterns of lung pathology we observed is that concurrent infection with CIV and SEZ caused obvious fibrosis lesions, especially in the preincubation group. Lung fibrosis is characterized by excessive deposition of extracellular matrix proteins, which results in impaired lung function and reduced gas exchange. TGF-*β* is considered a crucial mediator in tissue fibrosis and causes tissue scarring largely by activating its downstream Smad signaling [50]. Our data indicated that the transcription of TGF-*β* was significantly upregulated in the lungs after concurrent infection with CIV and SEZ. Then, the detection of phosphorylated Smad2/3 confirmed the activation of the Smad pathway, which corresponds with the severity of lung fibrosis. Additionally, our findings showed that meningitis was evident in simultaneously coinfected mice, and consistent with this, high viral and bacterial loads were measured, indicating that the blood‒brain barrier (BBB) might be disrupted. Senatorov et al. [51] verified that BBB dysfunction could be mediated by the activation of TGF-*β*. Therefore, upregulation of TGF-*β* in the brain might be involved in the occurrence of meningitis in affected mice. Previous studies have demonstrated that TGF-*β* can be activated by the NA protein during influenza virus infection through the removal of sialic acid moieties from latent TGF-*β* (LTGF-*β*, inactive precursor of TGF-*β*) to release the active TGF-*β* molecule [52, 53]. Based on these results, we hypothesize that concurrent infection with SEZ and CIV increases the activity of viral NA, which leads to activation of the host TGF-*β*/Smad pathway and subsequent lung fibrosis. In our study, lung fibrosis in the preincubation group was the most obvious, precisely matching the highest NA enzyme activity and TGF-*β* expression in this group, which further supports our hypotheses. Here, we can exclude the influence of bacterial/viral load on pulmonary fibrosis, since the bacterial/viral load is not related to fibrosis scoring in the lung. We found before Day 6 postinfection, the bacterial load of the BV group was significantly higher than that of the VB group and comparable to that of the CO group, but the fibrosis score of the BV group was comparable to that of the VB group and significantly lower than that of the CO group. Also, no significant difference was observed in viral load between the VB, BV, and CO groups, which did not match the degree of fibrosis.

During the 14-day observation period, notably, bacteria and viruses were consistently detected in the mice feces, although body weight and cytokine levels of mice indicated a gradual recovery. This appears contradictory as there is no end to the shedding of these two pathogens from the feces of the inoculated animals in clinical rehabilitation. A similar finding has been reported in a previous study, which indicates SEZ could be consistently shed from rectal secretions of healthy but inoculated pigs [54]. Also, a study on the influenza virus reported that viral RNA positivity had little correlation with gastrointestinal symptoms and outcomes [55]. Therefore, our results may indicate that fecal shedding of SEZ and CIV is not related to disease status. Nevertheless, the persistence of the pathogens in clinically healthy animals poses a constant threat of transmitting disease to previously uninfected herds.

In conclusion, determining the contribution of viral/bacterial coinfection to disease severity is highly complex due to the numerous confounding parameters that need to be considered. Many studies have been published on the coinfection of influenza viruses and bacteria, but they emphasize the consequences of viral infection, which is beneficial to bacterial growth and results in bacterial superinfection. However, our study demonstrated that primary CIV infection plays a positive role in the host's resistance to subsequent SEZ. More interestingly, simultaneous infection with CIV and SEZ after the *in vitro* preincubation exacerbated the coinfection. A better understanding of the combined effect of both pathogens on disease severity is required for the development of control strategies. This study also provides a new perspective for elucidating the pathogenesis of multiple pathogens in the same ecological niche.

## Figures and Tables

**Table 1:** Infection sequence of pathogens.

Group	−2 day (1st infection)	0 day (2nd infection)
B	PBS	SEZ
V	PBS	CIV
VB	CIV	SEZ
BV	SEZ	CIV
CO	PBS	Mix SEZ and CIV immediately upon infection
PRE	PBS	Preincubation SEZ with CIV for 12 hr in 25°C
PBS	PBS	PBS

**Table 2:** Significance analysis of weight change curve in mice.

Test details	Time points with significant difference (*P* < 0.05) (dpi)
B vs. PBS	6−14
V vs. PBS	5−14
VB vs. PBS	4−14
BV vs. PBS	4−14
CO vs. PBS	3−14
PRE vs. PBS	3−14
V and VB vs. B	None
BV vs. B	4, 5, 7–12, 14
CO vs. B	3–5, 7−14
PRE vs. B	2−14
VB vs. V	None
BV vs. V	5−14
CO vs. V	3−14
PRE vs. V	2−14
BV vs. VB	8−11
CO vs. VB	4, 6−13
PRE vs. VB	2, 5−14
CO and PRE vs. BV	None
CO vs. PRE	None

## Data Availability

All data generated or analyzed during this study are included in this published article and its additional information files.

## Supplementary Materials

Supplementary 1 The effect of preincubation on the counts of SEZ or CIV. The counts of SEZ (B and B^PRE^) and CIV (V and V^PRE^) in PBS and in preincubation with the other were quantified. *ns* indicates no significant difference.

Supplementary 2 NA activity of SEZ and/or CIV Prepare pathogens according to animal experiments and add corresponding pathogens according to the sequence of A's scheme. Fluorescence intensity at an excitation wavelength of 322 nm and emission wavelength of 450 nm was detected. The NA activity of each group was expressed as the fold change in fluorescence intensity with PBS.  ^*∗∗∗*^*P* < 0.001.
