# Supplementary material for: Pathogenic Characteristics of an Infection with Canine Influenza Virus and Streptococcus equi subsp. zooepidemicus Alone or in Combination in Mice
Source: Transbound Emerg Dis. 2024 Jan 17;2024:2237621. doi: 10.1155/2024/2237621 (PMC12016976; doi:10.1155/2024/2237621)
Supplement: Supplementary 1 — The effect of preincubation on the counts of SEZ or CIV. The counts of SEZ (B and BPRE) and CIV (V and VPRE) in PBS and in preincubation with the other were quantified. ns indicates no significant difference. [file 2237621.f1.docx]

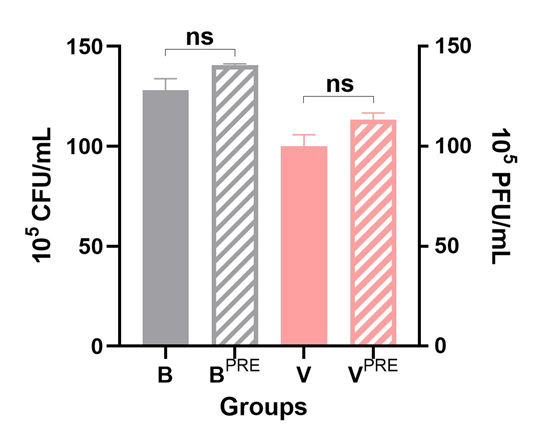


**Figure S1 The effect of preincubation on the counts of SEZ or CIV.** The counts of SEZ (B and B^PRE^) and CIV (V and V^PRE^) in PBS and in preincubation with the other were quantified. *ns* indicates no significant difference.
