# Supplementary material for: Pathogenic Characteristics of an Infection with Canine Influenza Virus and Streptococcus equi subsp. zooepidemicus Alone or in Combination in Mice
Source: Transbound Emerg Dis. 2024 Jan 17;2024:2237621. doi: 10.1155/2024/2237621 (PMC12016976; doi:10.1155/2024/2237621)
Supplement: Supplementary 2 — NA activity of SEZ and/or CIV Prepare pathogens according to animal experiments and add corresponding pathogens according to the sequence of A's scheme. Fluorescence intensity at an excitation wavelength of 322 nm and emission wavelength of 450 nm was detected. The NA activity of each group was expressed as the fold change in fluorescence intensity with PBS. ∗∗∗P < 0.001. [file 2237621.f2.docx]

**
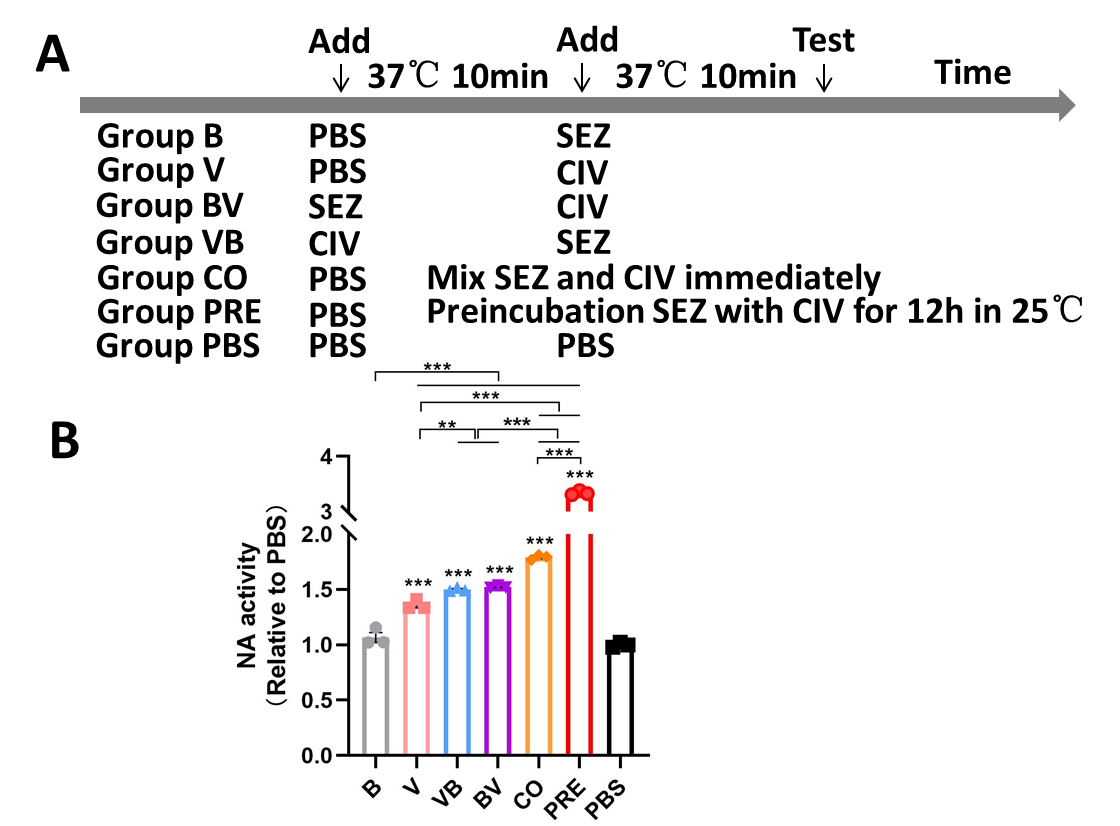
**

**Figure S2 NA activity of SEZ and/or CIV** Prepare pathogens according to animal experiments and add corresponding pathogens according to the sequence of A's scheme. Fluorescence intensity at an excitation wavelength of 322 nm and emission wavelength of 450 nm was detected. The NA activity of each group was expressed as the fold change in fluorescence intensity with PBS. ****P<*0.001.
